# Discovery of biomarkers in the psoriasis through machine learning and dynamic immune infiltration in three types of skin lesions

**DOI:** 10.3389/fimmu.2024.1388690

**Published:** 2024-05-13

**Authors:** Xiao Zhou, Han Zhou, Xin Luo, Rui-Fang Wu

**Affiliations:** ^1^ Department of Dermatology, The Second Xiangya Hospital of Central South University, Changsha, Hunan, China; ^2^ School of Mathematics and Statistics, Central South University, Changsha, Hunan, China

**Keywords:** psoriasis, atopic dermatitis, diagnosis genes, immune infiltration, machine learning algorithm

## Abstract

**Introduction:**

Psoriasis is a chronic skin disease characterized by unique scaling plaques. However, during the acute phase, psoriatic lesions exhibit eczematous changes, making them difficult to distinguish from atopic dermatitis, which poses challenges for the selection of biological agents. This study aimed to identify potential diagnostic genes in psoriatic lesions and investigate their clinical significance.

**Methods:**

GSE182740 datasets from the GEO database were analyzed for differential analysis; machine learning algorithms (SVM-RFE and LASSO regression models) are used to screen for diagnostic markers; CIBERSORTx is used to determine the dynamic changes of 22 different immune cell components in normal skin lesions, psoriatic non-lesional skin, and psoriatic lesional skin, as well as the expression of the diagnostic genes in 10 major immune cells, and real-time quantitative polymerase chain reaction (RT-qPCR) and immunohistochemistry are used to validate results.

**Results:**

We obtained 580 differentially expressed genes (DEGs) in the skin lesion and non-lesion of psoriasis patients, 813 DEGs in mixed patients between non-lesions and lesions, and 96 DEGs in the skin lesion and non-lesion of atopic dermatitis, respectively. Then 144 specific DEGs in psoriasis via a Veen diagram were identified. Ultimately, UGGT1, CCNE1, MMP9 and ARHGEF28 are identified for potential diagnostic genes from these 144 specific DEGs. The value of the selected diagnostic genes was verified by receiver operating characteristic (ROC) curves with expanded samples. The the area under the ROC curve (AUC) exceeded 0.7 for the four diagnosis genes. RT-qPCR results showed that compared to normal human epidermis, the expression of UGGT1, CCNE1, and MMP9 was significantly increased in patients with psoriasis, while ARHGEF28 expression was significantly decreased. Notably, the results of CIBERSORTx showed that CCNE1 was highly expressed in CD4+ T cells and neutrophils, ARHGEF28 was also expressed in mast cells. Additionally, CCNE1 was strongly correlated with IL-17/CXCL8/9/10 and CCL20. Immunohistochemical results showed increased nuclear expression of CCNE1 in psoriatic epidermal cells relative to normal.

**Conclusion:**

Based on the performance of the four genes in ROC curves and their expression in immune cells from patients with psoriasis, we suggest that CCNE1 possess higher diagnostic value.

## 1 Introduction

Psoriasis (PsO) and atopic dermatitis (AD) are common chronic inflammatory skin diseases (1). Typical psoriasis is characterized by the appearance of erythema, scaling, itching and pain on the surface of the skin, and its etiology is not yet fully understood but is thought to be related to genetics, the immune system and environmental factors (2, 3). AD, on the other hand, also has similar lesion characteristics to those of psoriatic lesions, and AD in particular can present as psoriasiform during the resistance phase (4). In addition to this, PsO manifests as eczema-like in the acute phase, there are also patients with psoriasis-like AD, and interestingly, there are even patients in whom PsO and AD can coexist (5, 6). Therefore, in view of the more complex manifestations of PsO and AD patients, there are really diagnostic difficulties in diagnosis and stereotyping (7). The correct diagnosis of these two diseases involves the correct selection and use of biologics, and the correct and effective use of biologics can realize its true treatment meaning (8, 9). Thus, for mixed patients or PsO patients whose lesions are difficult to distinguish from AD patients, more accurate diagnostic markers are needed to carry out differential diagnosis (10).

In this study, we carried out the GSE182740 datasets from the Gene Expression Omnibus (GEO) database for differential analysis, which included 20 AD (10 lesional and 10 non-lesional), 33 overlap phenotype of AD and PsO (17 lesional and 16 non-lesional), 16 PsO (9 lesional and 7 nonlesional), and 6 normal skin. By GEO2R analysis, we obtained 580 differentially expressed genes (DEGs) in the skin lesion and non-lesion of PsO patients, 813 DEGs in mixed patients between non-lesions and lesions, and 96 DEGs in the skin lesion and non-lesion of AD, respectively. The advantage of this kind of differential analysis between the RNA-seq of lesional and non-lesional skin in the same patient is that circumvent the problem of mismatch between healthy individuals and patients, thereby improving the accuracy of DEGs.

In recent years, machine learning algorithms have been increasingly used in the medical field to construct optimal classification models to filter out characterized variables (11). During our research, two machine algorithms, “least absolute shrinkage and selection operator” (LASSO) and “support vector machine - recursive feature elimination” (SVM-RFE), were utilized for diagnostic gene screening of differential genes (12, 13), and at the same time, CIBERSORT, as a widely used machine algorithm for classifying populations of immune cells (14), was applied to identify three types skin lesion (normal skin, psoriasis non-lesional skin and psoriasis lesional skin) and potential diagnosis genes of PsO associated with immune infiltration, and were verified by real-time quantitative polymerase chain reaction (RT-qPCR) experiments.

## 2 Materials and methods

### 2.1 Datasets information

The GSE182740 datasets obtained from http://www.ncbi.nlm.nih.gov/geo/was used in the present study. Specifically, the microarray data included 20 cases of AD (10 lesional and 10 non-lesional), 33 cases of AD and PsO with overlapping phenotypes (17 lesional and 16 non-lesional), 16 cases of PsO (9 lesional and 7 non-lesional), and 6 cases of normal skin (from GSE78097 datasets). Next, GEO2R was performed to obtain the DEGs between the non-lesional and lesional of AD, the non-lesional and lesional of PsO and the non-lesional and lesional of overlapping phenotypes in an attempt to reduce individual variation and improve the specificity of the DEGs. GEO2R is an interactive R-based online analysis tool that utilizes limma R packages to analyze the original data with the moderated t-statistic for significance analysis, and Benjamini & Hochberg false discovery rate method for applying *P-value adjustment* for multiple testing correction. In addition, normal skins and psoriatic lesions and psoriatic paraneoplastic lesions data were used for immune infiltration analysis with the aim of observing the immunological dynamics of three types of lesions ranging from mild to severe. *P < 0.05* and |log2 fold change (FC)| > 1 were used as threshold points for screening DEGs.

### 2.2 Functional pathway analyses

Sangerbox which is a comprehensive bioinformatics analysis platform (15) was performed on the 144 unique DEGs between psoriasis lesional (PsO-LS) and psoriasis non-lesional (PsO-NL) skin to uncover the possible gene functional annotation and pathway enrichment. *P value less than 0.05* and *adjusted P value less than 0.05* were selected as cutoff criteria.

### 2.3 Candidate gene biomarker identification

We employed two machine learning algorithms, namely the least absolute shrinkage and selection operator (LASSO) (16) and the support vector machine (SVM), to identify specific diagnostic gene biomarkers for psoriasis. As a regression analysis algorithm, LASSO is characterized by variable selection and regularization, which can avoid overfitting and enhance prediction accuracy. SVM is a supervised machine learning technique for classification and regression. The recursive feature elimination (RFE) algorithm can obtain the optimal variable combination that maximizes model performance. Combining these two techniques, we used the SVM-RFE algorithm to identify biomarkers with excellent discriminative abilities (17). The overlapping genes obtained from the above two algorithms were considered as candidate gene biomarkers. Specifically, LASSO was performed in conjunction with the glmnet R package to obtain the feature selections, important parameters are set as follows: standardize=Ture, alpha=1, family=gaussian, nfolds=3). In addition, we used mlbench and caret R packages to perform SVM-RFE, and all data were normalized prior to training the model. The 144 special DEGs between PsO-NL and PsO-LS, the results of LASSO and SVM-RFE, and the veen data of the two algorithms were provided respectively in the **Supplementary Tables 1**-**3**.

### 2.4 RNA extraction and real-time PCR

Skin lesion samples and peripheral blood were collected (samples were from the Second Xiangya Hospital of Central South University), and skin lesions were soaked in the dispase enzyme for 24h, isolation of epidermis for RNA extraction, peripheral blood was used to fractionate CD4+T cells. RNA was extracted using the TRIzol method. A reverse transcription kit produced by Takara in Japan was used to produce one nanogram of first-strand complementary DNA (cDNA). An ABI 7900 Real-Time PCR System was utilized for the execution of the real-time PCR. The relative expressions of were calculated using with a 2^−ΔΔCt^ method and normalized using GAPDH as an internal control. The primers used in this study were shown below **Table 1**.

**Table 1:** Real-time PCR primer sequences.

Gene	Forward primer	Reverse primer
UGGT1(Human)	TTCACCGCCAGCTTATATCAAAA	CCCCTGAACCTCATCAATAGGA
MMP9(Human)	GGGACGCAGACATCGTCATC	CGTCATCGTCGAAATGGGC
CCNE1(Human)	GCCAGCCTTGGGACAATAATG	CTTGCACGTTGAGTTTGGGT
ARHGEF28(Human)	AGGTGATGAAGTCTACGCTAACT	AGTGGCAGTGATTCCCTCTAT
RPLPO(Human)	GCAATGTTGCCAGTGTCTG	GCCTTGACCTTTTCAGCA

### 2.5 Diagnostic effectiveness examination

To further examine the validity of the prediction of diagnostic genes in PsO, we plotted receiver operating characteristic (ROC) curves on the mRNA expression data of normal samples and PsO patients in GSE182740, while calculating the area under the ROC curve (AUC) to determine the diagnostic validity of the diagnosis for PsO.

### 2.6 Immune infiltration analysis

To examine the expression of infiltrating immune cells in normal skin, psoriatic paralesional skin, and psoriatic lesions, we used a bioinformatics algorithm that identifies cell types by relative subsets of RNA transcripts(CIBERSORT, https://cibersortx.stanford.edu/). Putative immune cell abundance was calculated using a reference set of 22 immune cell species (LM22) and visualized using sangerbox. In addition, the expression of four diagnostic genes in immune cells was specifically analyzed. Correlation analysis was used to analyze the correlation between CCNE1 and inflammatory factors.

### 2.7 Immunohistochemistry

Skin lesions from patients with psoriasis vulgaris confirmed by histopathological examination, as well as skin tissues from normal individuals (all from the outpatient operating room of the Department of Dermatology, the Second Xiangya Hospital of Central South University) were collected after signing an informed consent form. Paraffin sections of the corresponding tissues were placed in an oven at 70°C for 2 hours, deparaffinized with turpentine for 30mins, then to 95%, 75%, 50%, deionized water for gradient rehydration, and autoclaved with antigenic restorative solution at pH 9.0 for 7mins, then cooled at room temperature. The slides were removed from the antigen repair solution and rinsed twice with TBST. Then 1 drop of 3% H_2_O_2_ was added to each section and incubated for 15 min at room temperature to block endogenous peroxidase activity. The sections were rinsed twice with TBST and 10ul cyclin E1 polyclonal antibody (proteintech, Cat No. 11554-1-AP) (dilution 1:400) was added to each section and incubated for 1 hour at room temperature. Rinse twice with TBST, add horseradish peroxidase (HRP)-labeled antibody to each section, and incubate for 30 min at room temperature. And then rinsed twice with TBST and observed under ordinary slice panoramic scanning (Hamamatsu Company, Japan). In multicolor immunofluorescence, add 10ul cyclin E1 polyclonal antibody (dilution 1:100), CD4 rabbit monoclonal antibody (RMA-0620, Fuzhou Maixin Biotechnology Company, China), Rb mAb to CD11b (Abcam, ab13357, 1:1000) incubated for 1 hour at room temperature to each tissue circle. After washing, use secondary antibody (Abcam, Anti-rabbit IgG H&L-HRP ab205722) (dilution 1:1000), apply and incubate for 20 minutes. After cleaning, add 10ul Opal 520, Opal 570 or Opal 650 respectively, to each tissue circle and incubate for 20 minutes, then 10ul DAPI (Servicebio Cat: G1012) for each tissue. After incubation for 20 minutes, Mantra pathological analysis system was used for analysis and photography (Akoya Company, USA).

### 2.8 Statistical analysis

Statistical analyzes used in this study used R (Version 4.3.1) and GraphPad Prism 8. Student’s unpaired t test was performed for normally distributed variables, while Mann-Whitney U test was performed for abnormally distributed variables. Differences were considered to be significant when the *P value was <0.05*.

## 3 Results

### 3.1 DEGs identification

**Figure 1:**
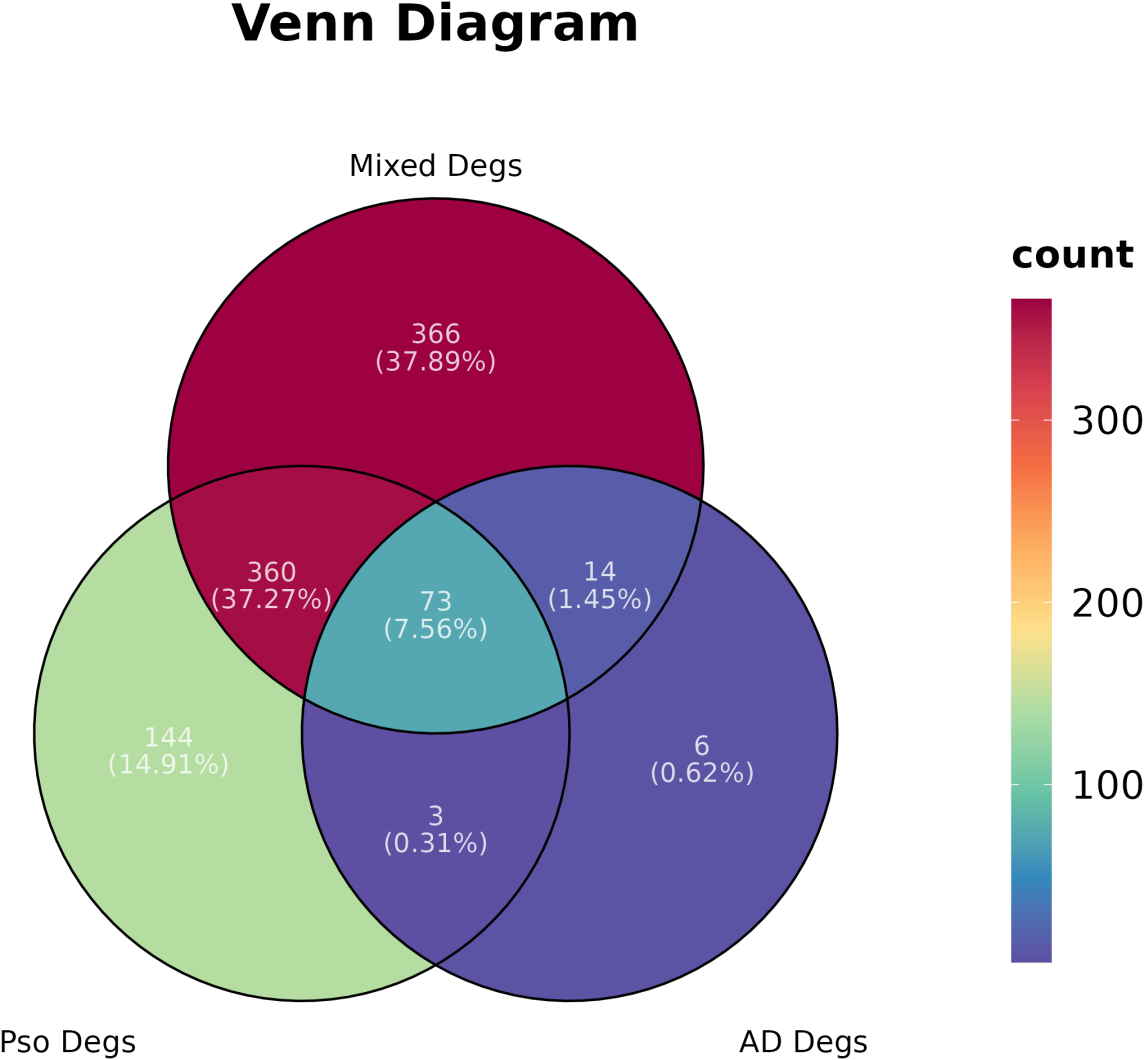
Differential gene expressions (Degs) network of psoriasis (Pso), atopic dermatitis (AD), and their Mixed in a Venn diagram.

In the present study, we conducted the GSE182740 for differential analysis on 9 PsO-LS and 7 PsO-NL skin biopsy samples, yielding 580 DEGs. Additionally, we obtained 96 DEGs from 10 atopic dermatitis non-lesional (AD-NL) and 10 atopic dermatitis lesional (AD-LS), as well as 813 DEGs from 16 mixed non-lesional (Mixed-NL) and 17 mixed lesional (Mixed-LS). As illustrated, we will primarily focus on the 580 DEGs between Pso-LS and Pso-NL, as well as the specific 144 DEGs that exclude those overlapping with the mixed DEGs and AD DEGs between non-lesional and lesional (**Figure 1**).

### 3.2 Functional enrichment analyses

**Figure 2:**
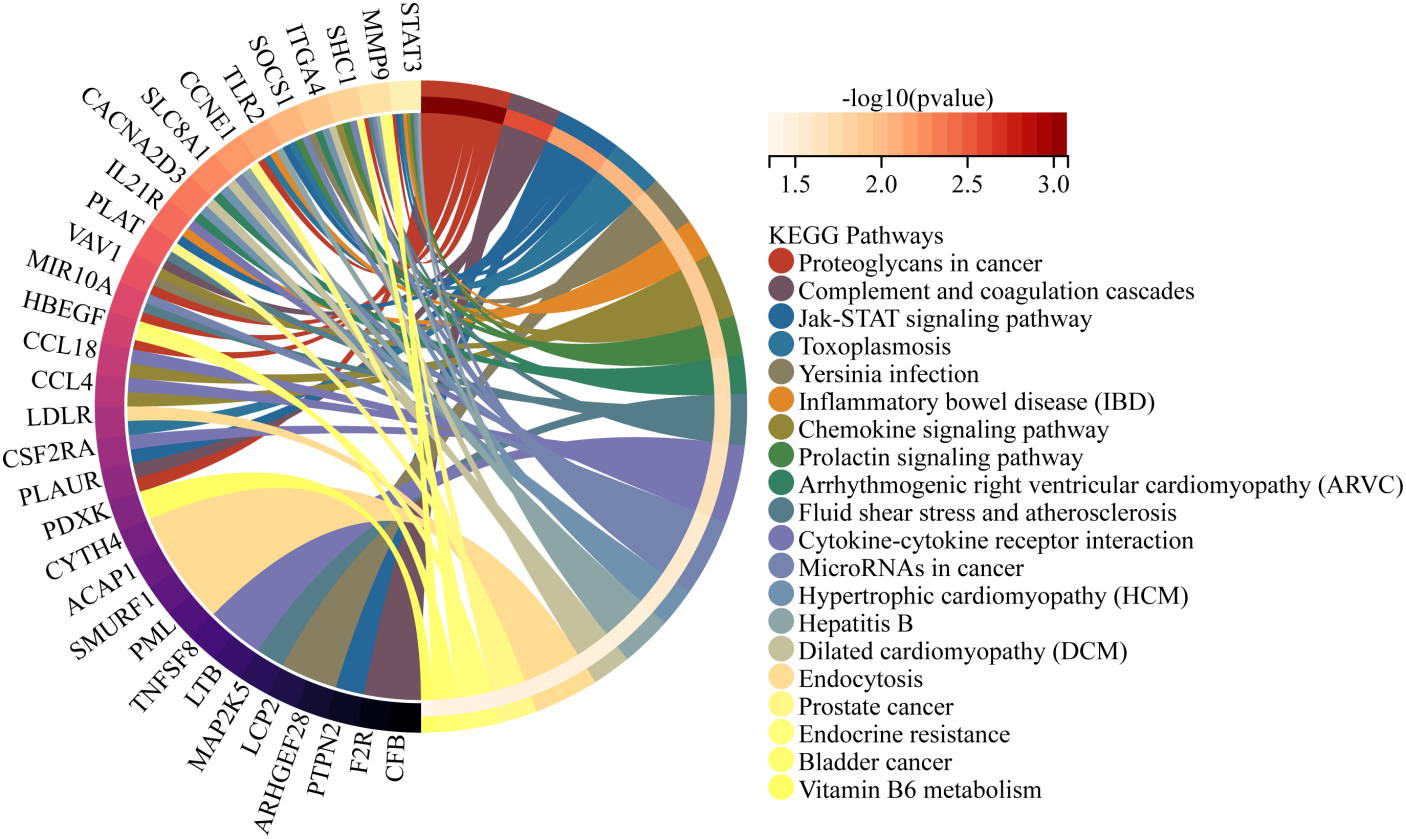
Functional enrichment analyses of the unique DEGs between PsO-NL and PsO-LS.

Moreover, we conducted functional enrichment analysis on the 144 unique DEGs between PsO-NL and PsO-LS. The top 20 pathways that were enriched for specific differential genes between PsO-NL and PsO-LS are shown in **Figure 2**. Proteoglycans in cancer, cytokine-cytokine receptor interaction, micro RNAs in cancer and JAK-STAT signaling pathway are the enrichment results for higher generatio.

### 3.3 Four DEGs were identified as specific diagnostic genes for psoriasis

**Figure 3:**
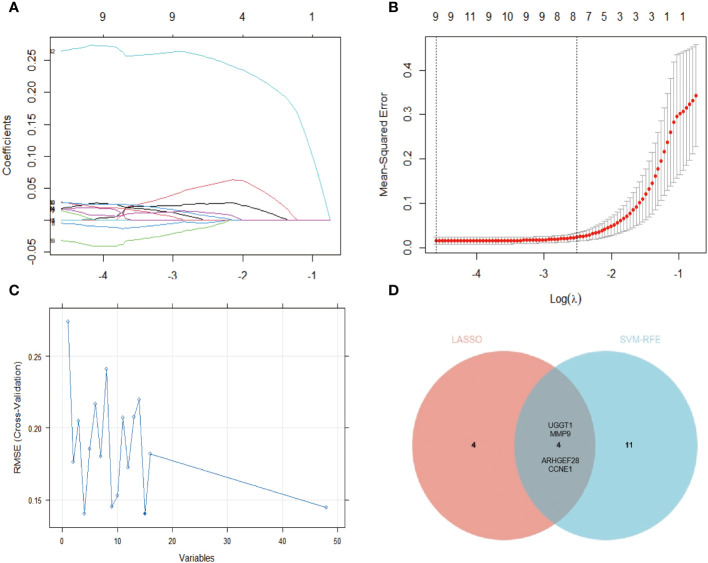
Identification of the gene biomarker for psoriasis diagnosis. **(A, B)** The LASSO logistic regression technique was utilized, the abscissa indicates the number of modeling genes corresponding to different (λ) values and eights genes with minimum lambda (λ) value were identified with 3-fold cross-validation. **(C)** A plot of gene biomarker selection by SVM-RFE. The blue dot indicates the best 15 genes. **(D)** The Venn data of the LASSO and SVM-RFE.

We employed LASSO and SVM-RFE algorithms to screen potential diagnostic genes from the above-mentioned 144 distinctive genes between PsO-NL and PsO-LS. Firstly, we utilized LASSO logistic regression and performed a penalty parameter tuning process with three-fold cross-validation, resulting in the identification of 8 genes as potential PsO biomarkers. Subsequently, the SVM-RFE algorithm identified 15 feature variables for diagnosing PsO. Ultimately, both algorithms selected 4 genes (UGGT1\MMP9\CCNE1\ARHGEF28) as the optimal candidate genes for characteristic gene expression (**Figure 3**).

### 3.4 The expressing patterns of diagnostic genes in psoriasis

**Figure 4:**
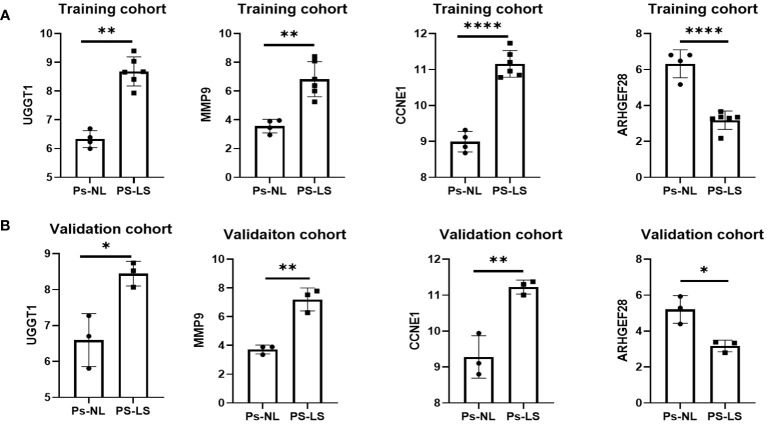
The expression level of UGGT1, MMP9, CCNE1, ARHGEF28 in training **(A)** and validation **(B)** cohorts. Ps-NL, psoriasis non-lesion; Ps-LS, psoriasis lesion. *P < 0.05, **P < 0.01, ****P < 0.0001.

To further confirm the expression of the optimal candidate genes in patients with PsO, we validated the training and validation cohorts. The results revealed that the expression of UGGT1, MMP9, and CCNE1 was significantly increased in PsO-LS compared with the PsO-NL, while ARHGEF28 exhibited a decreased status compared with the PsO-NL (**Figure 4**).

### 3.5 Diagnostic effectiveness examination via RT-qPCR & ROC

**Figure 5:**
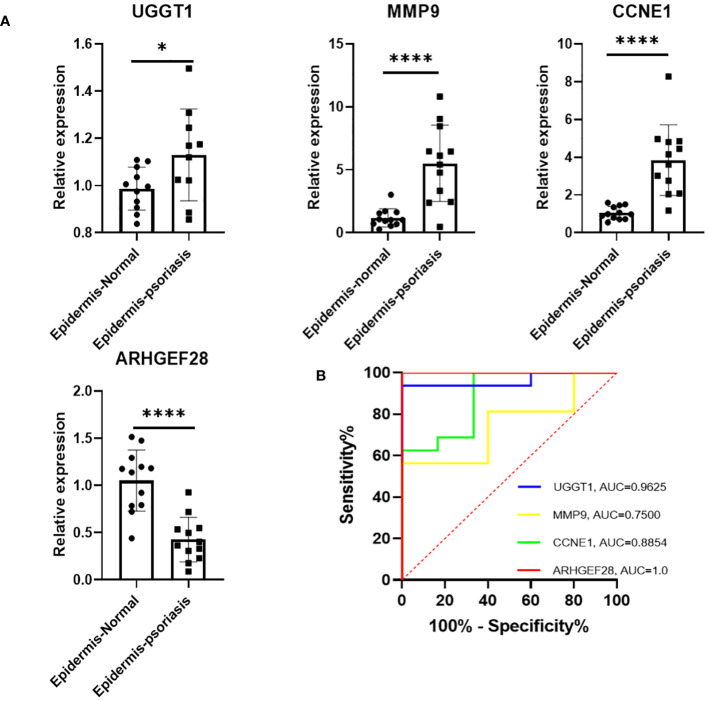
**(A)** Expression of four diagnostic genes in epidermis of PsO and normal human as revealed by RT-qPCR. **(B)** The determination of its diagnostic effectiveness between normal and psoriatic samples via ROC curves for UGGT1/MMP9/CCNE1/ARHGEF28. ROC, receiver operating characteristic. *P < 0.05, ****P < 0.0001.

The RT-qPCR results revealed compared with the normal epidemis, a significant upregulation of CCNE1, MMP9, and UGGT1 expression in psoriatic epidermis, in the contrast, a notable downregulation of ARHGEF28 in psoriatic epidermis. These findings were consistent with those observed in the cohort study. Moreover, ROC curves were generated for these four diagnosis genes to assess their discriminative power in distinguishing normal from psoriatic patient samples. The AUC of the ROC curve for the four potential diagnostic genes was 1.0 for ARHGEF28, 0.96 for UGGT1, 0.8854 for CCNE1, and 0.77 for MMP9 (**Figure 5**).

### 3.6 Association of three types lesions with the proportion of infiltrating immune cells

**Figure 6:**
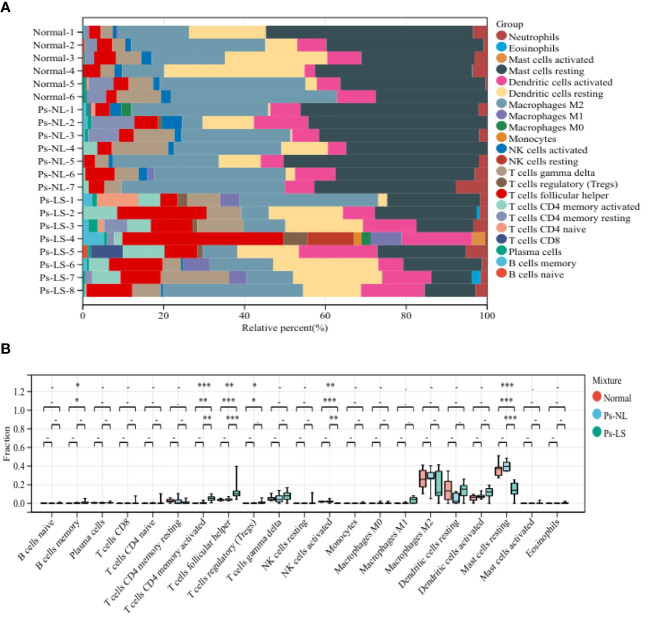
**(A)** A bar plot comparing the percentage of 22 different types of immune cells found infiltrating in three types lesions **(B)** three types lesions were compared using an histogram plot, which displayed the ratio differentiation of 22 distinct types of immune cells. Ps-NL, psoriasis non-lesion; Ps-LS, psoriasis lesion. * P < 0.05, **P < 0.01, ***P < 0.001.

We employed CIBERSORTx to analyze the composition of 22 immune cell types in 6 normal skins, 7 PsO-NL, and 8 PsO-LS within this dataset. First, in normal and PsO-LS as well as in PsO-NL and PsO-LS, significant differences were observed in CD4^+^ T cells memory activated, T cells follicular helper, NK cells activated, and mast cell resting populations. Additionally, meaningful statistical differences were exhibited by B cell and Tregs cell only in the normal skin and PsO-LS (**Figure 6**).

### 3.7 Four diagnostic genes in immune cell types and its correlation with chemokines

**Figure 7:**
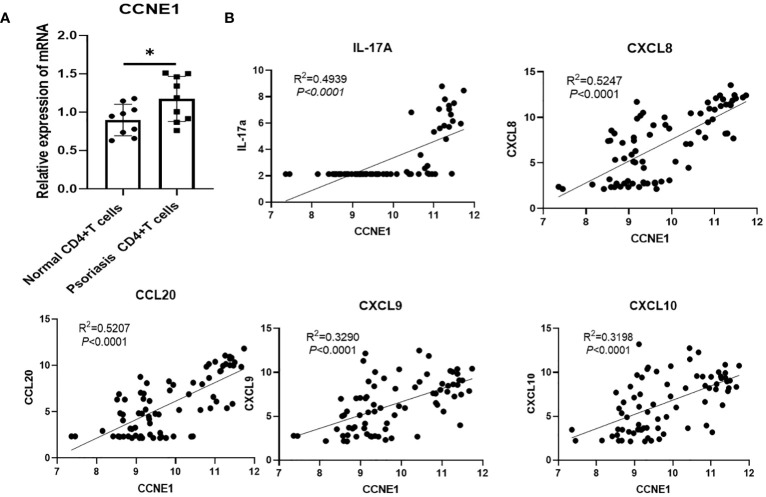
**(A)** Validation of CCNE1 in CD4+ T cells from controls and psoriasis patients **(B)** Correlation between CCNE1 and IL-17A, CXCL8/9/10 and CCL20. * P < 0.05.

Further, we analyzed the distribution of four diagnosis genes identified by LASSO and SVM-RFE in 10 major immune cell types. As shown in **Table 2**, we found that CCNE1 is highly expressed in CD4^+^ T cells and neutrophils in psoriatic lesions, at the same time, validation of the expression of CCNE1 in CD4+ T cells was performed by RT-qPCR (**Figure 7A**), and ARHGEF28 is highly expressed in mast cells. In addition, CCNE1 is closely related to the chemokines IL-17A, CXCL8/9/10 and CCL20 (**Figure 7B**).

**Table 2:** The expression of four diagnosis genes in 10 major immune cell types.

GeneSymbol	B cells	Plasma cells	T cells CD8	T cells CD4	NK cells	Monocytes	Dendritic cells	Mast cells	Eosinophils	Neutrophils
*MMP9*	-	-	-	-	0	-	-	0	-	-
*UGGT1*	-	-	-	-	-	-	-	-	-	-
*CCNE1*	-	-	-	12.26	0	-	-	0	-	12.38
*ARHGEF28*	-	-	-	-	-	-	-	10.88	0	-

Red highlight means that the gene is highly expressed in certain cell. Blue highlight means that the expression of certain gene is not obvious.

### 3.8 The elevation of CCNE1 in the psoriasis epidermis

**Figure 8:**
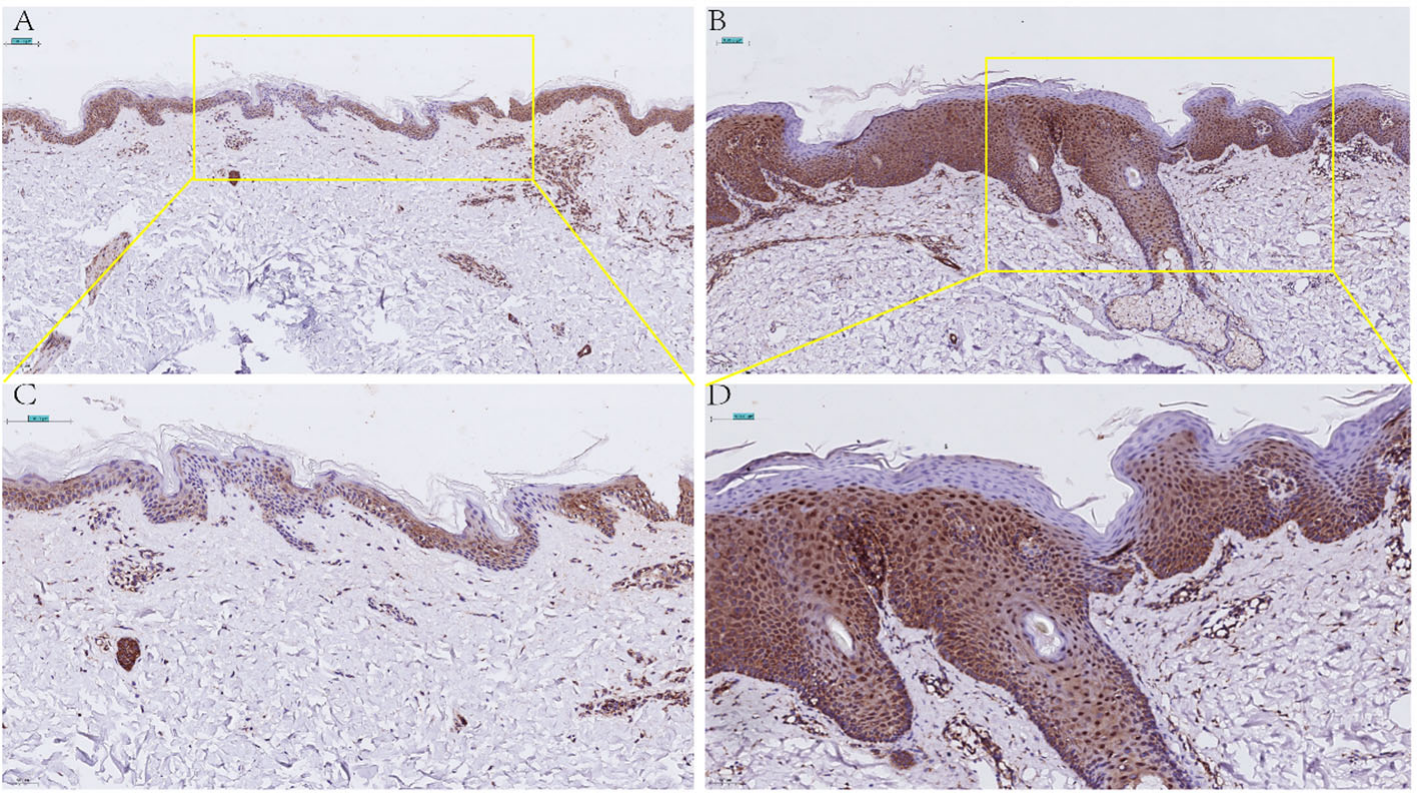
Immunohistochemical detection of CCNE1 expression in normal skin lesions (**A** 20X, **C** 40X) and psoriatic lesions (**B** 20X, **D** 40X).

Given the importance of CD4+T in psoriasis and the results of the four genes in immune cells, finally, CCNE1 was chosen for further validation. As shown, CCNE1 expression was significantly increased especially in the nucleus in psoriatic epidermis relative to normal controls (**Figure 8**).

### 3.9 The up-regulation of CCNE1 in immune cells in psoriatic skin lesions

**Figure 9:**
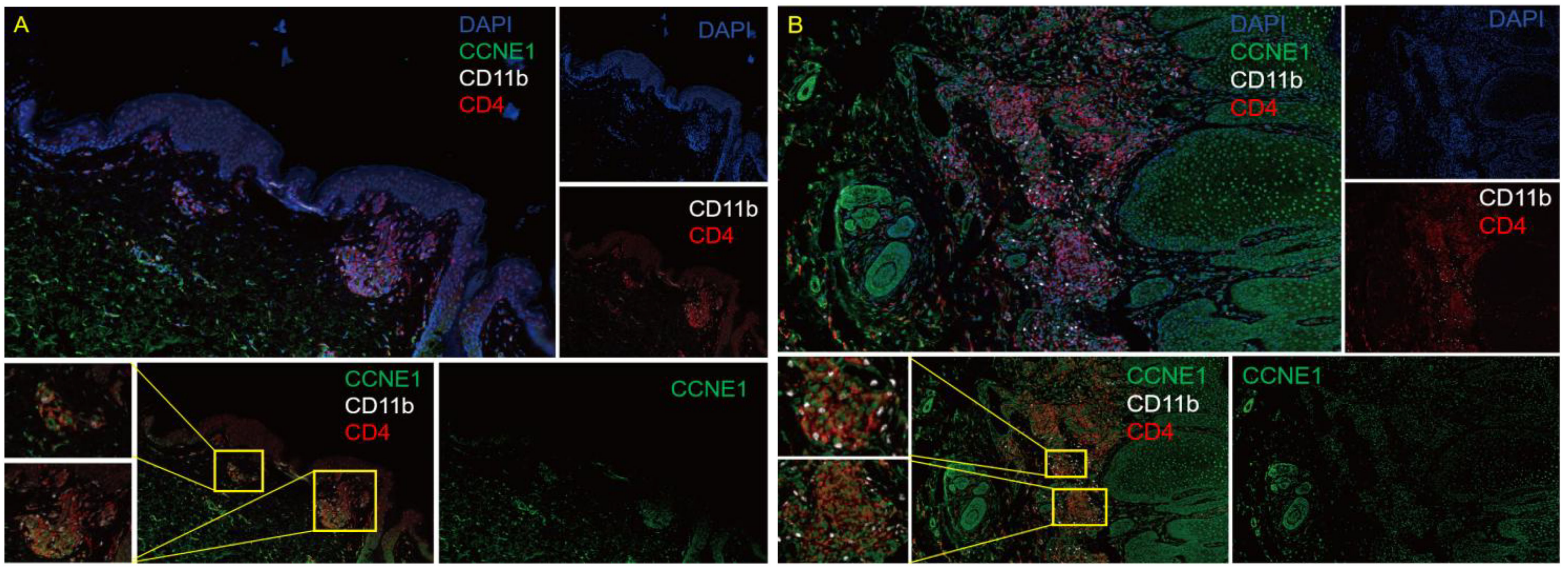
Multicolor immunofluorescence technology to detect the expression of CCNE1 in the CD4 + cells and neutrophils in normal human skin lesions **(A)**, 10X eyepiece, 10X objective) and PsO lesions **(B)**, 10X ocular lenses, 10X objective). Blue represents DAPI, green represents CCNE1, white represents CD11b positive, and red represents CD4+ positive.

Further, we used multi-color immunofluorescence technology to detect the expression of CCNE1 in CD4+ cells and neutrophils in PsO and normal control lesions. As shown in **Figure 9**, CCNE1 is expressed in both CD4+ cells and CD11b-labeled cells, and the expression in patients with PsO is significantly higher than that in normal people.

## 4 Discussion

Psoriasis vulgaris is a chronic inflammatory skin disease, affects approximately 125 million individuals globally, including 6.5 million in China (18, 19). Psoriasis with the character of recurrent episodes seriously affects the psychological and physical health of patients and aggravates the socio-economic burden and public health resources (20). Currently, biological agents show promising therapeutic effects for patients with typical psoriasis, however, patients with psoriasis-like atopic dermatitis often present challenges in differentiation from psoriasis (21), thus affecting the selection of biological agents and disease treatment progress.

Furthermore, we utilized the Venn diagram to exclude DEGs shared between the lesion and no-lesion of atopic dermatitis and mixed patients, obtaining 144 specific differential genes in psoriasis non-lesions and lesions. By employing the LASSO algorithm, we identified eight specific diagnostic genes, including TRIM13, CCNE1, UGGT1, MMP9, ARHGEF28, NAPG, CCNYL1, and NFASC. The SVM-RFE model selected 15 specific diagnostic genes. Taking the intersection of these two algorithms, we finally obtained four specific gene biomarkers for psoriasis diagnosis (UGGT1\MMP9\CCNE1\ARHGEF28).

To further explore the expression patterns of the four diagnostic genes in psoriasis patients, we analyzed the training and validation cohorts in the models. We observed a significant increase in UGGT1, MMP9, CCNE1 expression in psoriatic lesional skin compared with the psoriatic non-lesion, and a noteworthy decrease in ARHGEF28 expression in psoriatic lesional skin.

Next, we collected the epidermis of healthy volunteers and psoriasis, extracted RNA, and performed RT-qPCR for further validation. The results demonstrated a significant upregulation of UGGT1, MMP9, and CCNE1 expression in psoriatic patients, accompanied by a significant downregulation of ARHGEF28 expression. These findings were consistent with the algorithm-analysis outcomes. Additionally, we expanded the sample size and compared the diagnostic effectiveness with psoriatic and normal individuals. The ROC curve analysis revealed that the AUC of four diagnostic genes were greater than 0.7, with ARHGEF28 reaching 1.0, UGGT1 0.96, CCNE1 0.8854, and MMP9 0.77, indicating effective discriminative significance.

Additionally, we performed enrichment analysis on the 144 unique DEGs in psoriatic lesional and non-lesional skin. As shown in **Figure 2**, JAK-STAT signaling pathway, yersinia infection, inflammatory bowel diseases (IBD), cytokine-cytokine receptor interaction were performed in the psoriasis-specific enrichment results. As we all know, JAK-STAT signaling pathway is a key mediator underlying the immunopathogenesis of psoriasis, and its presence in our psoriasis-specific differential gene enrichment results provides a new piece of evidence that drugs targeting the JAK-STAT pathway for the treatment of psoriasis although they are still in the early stages of development (22, 23). Intriguingly, the results of the psoriasis-specific differential gene enrichment showed an association with IBD, which coincides with the results of the study in JAMA Dermatology (24), a cohort study that showed that patients with psoriasis had a 2.53-fold increased risk of Crohn’s disease and a 1.71-fold increased risk of ulcerative colitis. Furthermore, our results show that the genes mediating the development of IBD in psoriasis may be associated with the following genes CSF2RA/SOCS1/PTPN2/STAT3/IL21R (in the **Supplementary Table 4**), which has implications for the exploration of co-morbid targets in PsO combined with IBD. In addition, vitamin efficacy has been shown to be important in the treatment of psoriasis (25). We found that vitamin B6 metabolism occupies a place in the gene enrichment analysis specific to psoriasis, suggesting that vitamin B6 supplementation is an important part of vitamin therapy for psoriasis. It is worth mentioning that four diagnostic genes screened by our machine learning were shown to be associated with toxoplasmosis, yersinia infection, arrhythmogenic right ventricular cardiomyopathy (ARVC), and hepatitis B, dilated cardiomyopathy (DCM) disease in the enrichment results(in the **Supplementary Table 4**), which needs to draw attention to the importance of screening for cardiovascular and intestinal diseases in psoriasis patients as well as the development of therapeutic targets for patients with co-morbidities.

As we all know, psoriasis is an autoimmune inflammatory disease closely associated with the immune system. It is characterized by chronic inflammation and epidermal hyperplasia, with a substantial number of inflammatory and immune cells functioning simultaneously (26). CIBERSORTx is also a type of machine learning algorithm, also known as digital cytometry, which implements an inverse convolution approach through linear support vector regression (SVR) techniques that can analyze the gene expression patterns of cells in complex tissues to determine the cellular composition of the tissue (27, 28). In our research, we performed CIBERSORTx to analyze the infiltration of 22 immune cell types in the RNA-seq of normal skin lesions and the non-lesional and lesional skin regions of psoriatic patients. This three types skin biopsy ranged from mild to severe allowed us to dynamically observe the immune cell dynamics in the three distinct stages of inflammation lesions. As shown in **Figure 6**, in adaptive immunization, we first observed a positive correlation between T cells CD4^+^ memory activated and T cells CD4^+^ follicular helper in normal skin lesions, non-lesional areas in psoriatic patients, and lesional areas in psoriatic patients, therefore, we speculate that T cells CD4^+^ memory activated and T cells CD4^+^ follicular helper might play an indispensable regulatory role throughout the whole process of change from mild to severe process of psoriasis-like skin lesions. Numerous studies on the immune pathogenesis of psoriasis have focused on T lymphocytes, and their importance in this disease has been widely acknowledged (29, 30). Additionally, we found activated dendritic cells (DCs) showed an upward trend in normal individuals, psoriatic non-lesional areas, and psoriatic lesions, which is consistent with the conclusion that DCs are increased in psoriatic lesions and are thought to contribute to the formation of a T-cell response (31). Interestingly, B cells memory and Tregs cells exhibited significant differences only in normal skins and psoriatic lesional areas, suggesting that B cells memory or Tregs might carry a more prominent role in the later stages of lesion progression.

On the other hand, in the innate immune response, the results of immune infiltration showed a significant decrease in activated NK cells in psoriatic lesional skin compared to normal skin and non-lesional skin in psoriatic patients. This phenomenon is consistent with the reduced number of NK cells in the peripheral blood of psoriatic patients, but there are few reports on the changes in the proportion of NK cells in lesions, based on the results of this study, we prefer that NK cells activation can maintain skin immune homeostasis (32, 33). Also interestingly, the results of CIBERSORTx add a new piece of evidence for the role of mast cells in the progression of psoriatic skin lesions. And our three skin lesion immuno dynamic infiltration results showed that resting state mast cells showed a gradual decrease in skin lesions in psoriasis patients compared with non-lesional skin in psoriasis patients, suggesting that resting mast cells may play an important role in the maintenance of skin homeostasis. In addition, mast cells may activate and degranulate during the progression of psoriasis, ultimately leading to chronic inflammation (34). In brief, we suggest that the reduction of resting mast cells may cause disturbances in skin homeostasis and thus exacerbate the changes in the lesions. Taken together with the reduction of NK cells and mast cells in psoriatic lesions, we hypothesize that innate immunity plays an important role in skin homeostasis, and that the progression of lesions toward psoriasis-like conditions may be associated with a reduction in innate immune cell function.

Further we analyzed the expression of the four diagnostic genes screened by the two machine algorithms in 10 major immune cells and found that CCNE1 was elevated in CD4^+^ T cells as well as neutrophils in psoriasis lesions, and ARHGEF28 was elevated in mast cells. UGGT1 and MMP9 were not significantly enriched in 10 major immune cells. It is worth mentioning that we verified the expression of CCNE1 in CD4+ cells and neutrophils which located in the lesion through multicolor immunofluorescence technology. Actually, psoriasis is a T-cell mediated autoimmune skin disease, IFN-gama, TNF-α, IL-17A, CXCLs, CCLs, and antimicrobial peptide secreted by immune cells or keratinocytes undergo important functions in the crosstalk between keratinocyte and immune cells in the pathogenesis of psoriasis (35–38), thus, in view of the results that we validated in CD4^+^ T cell with CCNE1, we pay more attention to the correlation analysis between CCNE1 and inflammatory mediators mentioned above, and the results showed that CCNE1 was significantly correlated with IL-17A/CXCL8/CXCL9/CXCL10/CCL20 (P<0.0001, R^2^>0.3). Indeed, CCNE1 is a positive regulator of the cell cycle, which can activate cyclin-dependent kinases 2 (CDK2) to regulate the G1-S transition of the cell division cycle, and plays an important role in the regulation of cell cycle progression and cell proliferation (39).To further investigate the role of CCNE1 in psoriasis, we performed immunohistochemical experiments, which showed that CCNE1, as a nuclear protein, was increased in the nuclei of cells in the epidermis of psoriasis relative to normal human skin. It has been demonstrated that IL-17A stimulates keratinocytes to upregulate the cell cycle-related genes, such as CCNE1, which made contribution to epidermal KC proliferation (40), therefore we hypothesize that enhanced IL-17 may contribute to the elevated CCNE1 in epidermal cells of psoriasis patients. And studies have shown that CXCL8 could recruit neutrophils and CCL20 chemotactically attracts IL-17A-producing immune cells, further creating an IL-17A-rich environment and accelerating the pathological progression of psoriasis (41). In addition, elevated expression of CXCL9 and CXCL10 in epidermal keratinocytes may induce type 1 T cells to migrate from the dermis to the epidermis and trigger inflammation in the skin (42–44). Nevertheless, whether CCNE1 is able to regulate the expression of CXCL8/9/10,CCL20, and thus mediate the above mentioned progression of psoriasis pathological mechanisms is not enough to rely only on correlation analysis, more experiments should be performed. In addition, CCNE1 was also highly expressed in CD4+T cells as well as neutrophils in our immune infiltration results, although the mechanism of CCNE1 elevation in CD4+ as well as neutrophils has indeed been rarely studied, Neutrophil traps and TH17 release of IL-17 in psoriatic lesions is common (45), and based on the fact that IL-17 induces epidermal cells to produce CCNE1 expression, we speculate whether there is a similar stimulatory effect in CD4+T cells as well as neutrophils, but more studies are needed to confirm this. Based on existing research, and we found that CCNE1 may not only play a role in skin lesions through the cell cycle, but may also intervene in the progression of psoriasis lesions by modulating immune cells as well as secretion of inflammatory factors.

Overall, we circumvented the problem of mismatch between healthy individuals and patients by differential gene analysis of lesions and non-lesional areas in psoriasis, atopic dermatitis, and overlapping patients and obtained 144 psoriasis-specific differential genes, then four diagnostic genes were then screened by two machine algorithms, and finally the dynamics of the immune cells in the three degrees of lesions were analyzed by using a reverse convolutional algorithm. The manifestations of the four diagnostic genes in immune cells were also specifically analyzed, and we also performed RT-qPCR and immunohistochemistry for experimental validation, and the results confirmed to be consistent with those obtained by the machine analysis algorithms, which suggests that the application of machine algorithms in the medical field is reliable and valuable. Finally, we obtained four diagnostic genes with research value, and we have a greater interest in CCNE1, in view of the AUC of ROC and their expression in immune cells. However, in the future, we should pay more effort into the validation and investigation of the specific regulatory mechanisms of CCNE1 with CXCL8/9/10 and CCL20, and whether IL-17 can stimulate CCNE1 expression in keratinocytes, CD4+T cells and neutrophils in the pathogenesis of psoriasis, in addition to this, expanded samples are also needed to further improve the sensitivity of the diagnostic genes.

## Data availability statement

The datasets presented in this study can be found in online repositories. The names of the repository/repositories and accession number(s) can be found in the article/**Supplementary Material**.

## Ethics statement

The studies involving humans were approved by Institutional Review Boards of Hanyang University, Seoul, Korea, and Rockefeller University, New York, NY, USA. Written informed consent was obtained from all patients. The studies were conducted in accordance with the local legislation and institutional requirements. Written informed consent for participation was not required from the participants or the participants’ legal guardians/next of kin in accordance with the national legislation and institutional requirements.

## Author contributions

XZ: Conceptualization, Methodology, Supervision, Visualization, Data curation, Writing – original draft, Writing – review & editing. HZ: Conceptualization, Methodology, Supervision, Visualization, Data curation, Formal analysis, Writing – review & editing. XL: Conceptualization, Validation, Visualization, Writing – review & editing. R-FW: Conceptualization, Funding acquisition, Project administration, Writing – review & editing.
